# 4,4′-Trimethyl­enedipiperidiniumbenzene-1,4-dicarboxyl­ate

**DOI:** 10.1107/S1600536809042901

**Published:** 2009-10-23

**Authors:** Feng-Shuen Tseng, Chia-Her Lin

**Affiliations:** a Department of Chemistry, Chung-Yuan Christian University, Chung-Li 320, Taiwan

## Abstract

The hydro­thermal reaction of benzene-1,4-dicarboxylic acid and 4,4′-trimethyl­ene dipiperidine leads to the formation of the title compound, C_13_H_28_N_2_^2+^·C_8_H_4_O_4_^2−^. The anion is located on a center of inversion whereas the cation is positioned on a twofold rotation axis. In the crystal structure, the anions and cations are linked by N—H⋯O and N—H⋯(O,O) hydrogen bonds.

## Related literature

For general background, see: Moulton & Zaworotko (2001 ▶).
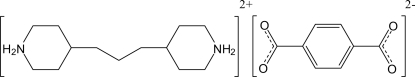


## Experimental

### 

#### Crystal data

C_13_H_28_N_2_^2+^·C_8_H_4_O_4_^2−^
*M* *_r_* = 376.49
Monoclinic, 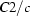

*a* = 20.2902 (18) Å
*b* = 8.4534 (8) Å
*c* = 11.8815 (9) Å
β = 108.610 (3)°
*V* = 1931.4 (3) Å^3^
*Z* = 4
Mo *K*α radiation
μ = 0.09 mm^−1^
*T* = 295 K
0.25 × 0.20 × 0.20 mm

#### Data collection

Bruker APEXII CCD diffractometer
Absorption correction: multi-scan (*SADABS*; Bruker, 2008 ▶) *T*_min_ = 0.978, *T*_max_ = 0.982
9090 measured reflections
2286 independent reflections
1073 reflections with *I* > 2σ(*I*)
*R*_int_ = 0.114

#### Refinement

*R*[*F* ^2^ > 2σ(*F* ^2^)] = 0.046
*wR*(*F* ^2^) = 0.095
*S* = 0.82
2286 reflections
124 parameters
H-atom parameters constrained
Δρ_max_ = 0.18 e Å^−3^
Δρ_min_ = −0.20 e Å^−3^

### 

Data collection: *APEX2* (Bruker, 2008 ▶); cell refinement: *SAINT-Plus* (Bruker, 2008 ▶); data reduction: *SAINT-Plus*; program(s) used to solve structure: *SHELXS97* (Sheldrick, 2008 ▶); program(s) used to refine structure: *SHELXL97* (Sheldrick, 2008 ▶); molecular graphics: *DIAMOND* (Brandenburg, 2009 ▶); software used to prepare material for publication: *SHELXTL* (Sheldrick, 2008 ▶).

## Supplementary Material

Crystal structure: contains datablocks I. DOI: 10.1107/S1600536809042901/nc2162sup1.cif

Structure factors: contains datablocks I. DOI: 10.1107/S1600536809042901/nc2162Isup2.hkl

Additional supplementary materials:  crystallographic information; 3D view; checkCIF report

## Figures and Tables

**Table 1:** Hydrogen-bond geometry (Å, °)

*D*—H⋯*A*	*D*—H	H⋯*A*	*D*⋯*A*	*D*—H⋯*A*
N1—H1*N*1⋯O1^i^	0.90	1.91	2.7958 (19)	169
N1—H2*N*1⋯O2	0.90	1.82	2.7213 (18)	174
N1—H2*N*1⋯O1	0.90	2.61	3.1528 (19)	120

Symmetry code: (i) 
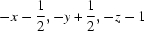
.

**Fig. 1.**
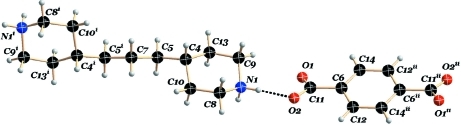
Crystal structure of the title compound with labeling and displacement ellipsoids drawn at the 50% probability level for non-H atoms. The hydrogen bonding is indicated as a dashed line. Symmetry codes: (i) -1 - x, y, -1/2 - z; (ii) -x, -y, -1 - z.].

## supplementary crystallographic information

### Comment

Crystals of the title compound were obtained by accident during our studies on the synthesis and structural characterization of coordination polymers. For their identification the crystal structure was determined.

The asymmetric unit consists of benzene-1,4-dicarboxylate anions and 4,4'-trimethylene dipiperidine cations each of them located in special positions. The anions are positioned on centers of inversion, whereas the cations are located on a 2-fold rotation axis which goes through the central C atom C7.

In the crystal structure the anions and cations are connected via N-H···O hydrogen bonding between the amino H atoms and the carboxylate oxygen atoms (Table 1).

### Experimental

The title compound was prepared by the reaction of 4,4'-trimethylenedipiperidine (0.0840 g, 0.4 mmol) and benzene-1,4-dicarboxylic acid (0.1661 g, 2.0 mmol) in H_2_O (1.0 ml) and CH_3_CN (5.0 ml). The mixture was heated to 393 K for 2 days in a Teflon-lined autoclave with an internal volume of 23 ml followed by slow cooling at 6 K/h to room temperature. The title compound was obtained as colorless crystals.

### Refinement

All hydrogen atoms were placed in idealized positions and constrained to ride on their parent atoms, with C—H = 0.93, 0.96, and 0.97 Å and *U*_iso_(H) = 1.2 *U*_eq_(C) and N—H = 0.90 Å and *U*_iso_(H) = 1.2 *U*_eq_(N).

### Crystal data

**Table:**

C_13_H_28_N_2_^2+^·C_8_H_4_O_4_^2^^−^	*F*(000) = 816
*M**_r_* = 376.49	*D*_x_ = 1.295 Mg m^−^^3^
Monoclinic, *C*2/*c*	Mo *K*α radiation, λ = 0.71073 Å
Hall symbol: -C 2yc	Cell parameters from 2292 reflections
*a* = 20.2902 (18) Å	θ = 3.0–26.1°
*b* = 8.4534 (8) Å	µ = 0.09 mm^−^^1^
*c* = 11.8815 (9) Å	*T* = 295 K
β = 108.610 (3)°	Columnar, colourless
*V* = 1931.4 (3) Å^3^	0.25 × 0.20 × 0.20 mm
*Z* = 4	

### Data collection

**Table:**

Bruker APEXII CCD diffractometer	2286 independent reflections
Radiation source: fine-focus sealed tube	1073 reflections with *I* > 2σ(*I*)
graphite	*R*_int_ = 0.114
Detector resolution: 8.3333 pixels mm^-1^	θ_max_ = 28.3°, θ_min_ = 2.1°
φ and ω scans	*h* = −25→26
Absorption correction: multi-scan (*SADABS*; Bruker, 2008)	*k* = −11→9
*T*_min_ = 0.978, *T*_max_ = 0.982	*l* = −10→14
9090 measured reflections	

### Refinement

**Table:**

Refinement on *F*^2^	Secondary atom site location: difference Fourier map
Least-squares matrix: full	Hydrogen site location: inferred from neighbouring sites
*R*[*F*^2^ > 2σ(*F*^2^)] = 0.046	H-atom parameters constrained
*wR*(*F*^2^) = 0.095	*w* = 1/[σ^2^(*F*_o_^2^) + (0.0287*P*)^2^] where *P* = (*F*_o_^2^ + 2*F*_c_^2^)/3
*S* = 0.82	(Δ/σ)_max_ < 0.001
2286 reflections	Δρ_max_ = 0.18 e Å^−^^3^
124 parameters	Δρ_min_ = −0.20 e Å^−^^3^
0 restraints	Extinction correction: *SHELXL*, Fc^*^=kFc[1+0.001xFc^2^λ^3^/sin(2θ)]^-1/4^
Primary atom site location: structure-invariant direct methods	Extinction coefficient: 0.0026 (5)

### Special details

**Table:**

Geometry. All esds (except the esd in the dihedral angle between two l.s. planes) are estimated using the full covariance matrix. The cell esds are taken into account individually in the estimation of esds in distances, angles and torsion angles; correlations between esds in cell parameters are only used when they are defined by crystal symmetry. An approximate (isotropic) treatment of cell esds is used for estimating esds involving l.s. planes.
Refinement. Refinement of F^2^ against ALL reflections. The weighted R-factor wR and goodness of fit S are based on F^2^, conventional R-factors R are based on F, with F set to zero for negative F^2^. The threshold expression of F^2^ > 2sigma(F^2^) is used only for calculating R-factors(gt) etc. and is not relevant to the choice of reflections for refinement. R-factors based on F^2^ are statistically about twice as large as those based on F, and R- factors based on ALL data will be even larger.

### Fractional atomic coordinates and isotropic or equivalent isotropic displacement parameters (Å^2^)

**Table:**

	*x*	*y*	*z*	*U*_iso_*/*U*_eq_	
N1	−0.28726 (7)	0.06898 (17)	−0.42869 (12)	0.0323 (4)	
H1N1	−0.3055	0.1498	−0.4782	0.039*	
H2N1	−0.2517	0.0285	−0.4493	0.039*	
C4	−0.38067 (9)	0.0846 (2)	−0.28506 (14)	0.0280 (4)	
H4A	−0.3658	−0.0015	−0.2272	0.034*	
C5	−0.44100 (9)	0.1711 (2)	−0.26231 (15)	0.0308 (5)	
H5A	−0.4226	0.2327	−0.1902	0.037*	
H5B	−0.4601	0.2450	−0.3269	0.037*	
C7	−0.5000	0.0702 (3)	−0.2500	0.0322 (7)	
H7A	−0.4829	0.0036	−0.1814	0.039*	
C8	−0.34100 (9)	−0.0545 (2)	−0.44141 (16)	0.0343 (5)	
H8A	−0.3213	−0.1429	−0.3896	0.041*	
H8B	−0.3569	−0.0929	−0.5226	0.041*	
C9	−0.26075 (9)	0.1290 (2)	−0.30457 (15)	0.0379 (5)	
H9A	−0.2259	0.2099	−0.2988	0.045*	
H9B	−0.2390	0.0432	−0.2515	0.045*	
C10	−0.40175 (9)	0.0137 (2)	−0.40950 (15)	0.0310 (5)	
H10A	−0.4357	−0.0692	−0.4152	0.037*	
H10B	−0.4238	0.0950	−0.4667	0.037*	
C13	−0.31977 (9)	0.1974 (2)	−0.26785 (16)	0.0354 (5)	
H13A	−0.3364	0.2927	−0.3136	0.042*	
H13B	−0.3020	0.2274	−0.1848	0.042*	
O1	−0.13904 (7)	0.19495 (16)	−0.41743 (12)	0.0428 (4)	
O2	−0.18035 (7)	−0.03599 (17)	−0.50059 (12)	0.0522 (4)	
C6	−0.06382 (9)	0.0291 (2)	−0.48175 (15)	0.0290 (4)	
C11	−0.13273 (10)	0.0646 (2)	−0.46455 (16)	0.0346 (5)	
C12	−0.05893 (10)	−0.0776 (2)	−0.56701 (16)	0.0345 (5)	
H12A	−0.0986	−0.1304	−0.6131	0.041*	
C14	−0.00395 (10)	0.1067 (2)	−0.41537 (15)	0.0345 (5)	
H14A	−0.0062	0.1796	−0.3580	0.041*	

### Atomic displacement parameters (Å^2^)

**Table:**

	*U*^11^	*U*^22^	*U*^33^	*U*^12^	*U*^13^	*U*^23^
N1	0.0262 (9)	0.0356 (10)	0.0398 (10)	0.0020 (7)	0.0173 (7)	0.0003 (7)
C4	0.0246 (10)	0.0315 (11)	0.0309 (11)	−0.0002 (9)	0.0130 (8)	0.0033 (8)
C5	0.0279 (11)	0.0354 (12)	0.0339 (11)	−0.0001 (9)	0.0164 (8)	−0.0012 (8)
C7	0.0281 (15)	0.0353 (17)	0.0388 (16)	0.000	0.0186 (12)	0.000
C8	0.0299 (11)	0.0354 (12)	0.0406 (12)	−0.0022 (9)	0.0154 (9)	−0.0048 (9)
C9	0.0271 (11)	0.0500 (13)	0.0378 (12)	−0.0074 (10)	0.0121 (9)	−0.0076 (9)
C10	0.0233 (10)	0.0347 (12)	0.0371 (11)	−0.0039 (9)	0.0125 (8)	−0.0012 (9)
C13	0.0305 (12)	0.0445 (13)	0.0342 (11)	−0.0034 (10)	0.0148 (9)	−0.0055 (9)
O1	0.0409 (9)	0.0385 (9)	0.0579 (9)	0.0085 (7)	0.0281 (7)	0.0027 (7)
O2	0.0315 (9)	0.0544 (10)	0.0797 (11)	−0.0039 (8)	0.0303 (7)	−0.0104 (8)
C6	0.0255 (11)	0.0287 (11)	0.0377 (11)	0.0039 (9)	0.0170 (8)	0.0077 (9)
C11	0.0306 (12)	0.0374 (13)	0.0417 (12)	0.0074 (11)	0.0199 (9)	0.0108 (10)
C12	0.0265 (11)	0.0358 (13)	0.0421 (12)	0.0002 (9)	0.0123 (9)	−0.0004 (9)
C14	0.0355 (12)	0.0336 (13)	0.0382 (12)	0.0044 (10)	0.0173 (9)	−0.0007 (9)

### Geometric parameters (Å, °)

**Table:**

N1—C8	1.482 (2)	C9—C13	1.514 (2)
N1—C9	1.488 (2)	C9—H9A	0.9700
N1—H1N1	0.9000	C9—H9B	0.9700
N1—H2N1	0.9000	C10—H10A	0.9700
C4—C13	1.522 (2)	C10—H10B	0.9700
C4—C5	1.522 (2)	C13—H13A	0.9700
C4—C10	1.525 (2)	C13—H13B	0.9700
C4—H4A	0.9800	O1—C11	1.261 (2)
C5—C7	1.515 (2)	O2—C11	1.255 (2)
C5—H5A	0.9700	C6—C12	1.383 (2)
C5—H5B	0.9700	C6—C14	1.384 (2)
C7—C5^i^	1.514 (2)	C6—C11	1.506 (2)
C7—H7A	0.9598	C12—C14^ii^	1.379 (2)
C8—C10	1.514 (2)	C12—H12A	0.9300
C8—H8A	0.9700	C14—C12^ii^	1.379 (2)
C8—H8B	0.9700	C14—H14A	0.9300
			
C8—N1—C9	111.26 (13)	C13—C9—H9A	109.6
C8—N1—H1N1	109.4	N1—C9—H9B	109.6
C9—N1—H1N1	109.4	C13—C9—H9B	109.6
C8—N1—H2N1	109.4	H9A—C9—H9B	108.1
C9—N1—H2N1	109.4	C8—C10—C4	113.16 (14)
H1N1—N1—H2N1	108.0	C8—C10—H10A	108.9
C13—C4—C5	109.83 (14)	C4—C10—H10A	108.9
C13—C4—C10	109.95 (14)	C8—C10—H10B	108.9
C5—C4—C10	111.61 (14)	C4—C10—H10B	108.9
C13—C4—H4A	108.5	H10A—C10—H10B	107.8
C5—C4—H4A	108.5	C9—C13—C4	113.86 (15)
C10—C4—H4A	108.5	C9—C13—H13A	108.8
C7—C5—C4	116.86 (15)	C4—C13—H13A	108.8
C7—C5—H5A	108.1	C9—C13—H13B	108.8
C4—C5—H5A	108.1	C4—C13—H13B	108.8
C7—C5—H5B	108.1	H13A—C13—H13B	107.7
C4—C5—H5B	108.1	C12—C6—C14	118.11 (16)
H5A—C5—H5B	107.3	C12—C6—C11	121.05 (17)
C5^i^—C7—C5	111.4 (2)	C14—C6—C11	120.80 (17)
C5^i^—C7—H7A	109.3	O2—C11—O1	124.56 (18)
C5—C7—H7A	109.3	O2—C11—C6	117.75 (18)
N1—C8—C10	109.74 (15)	O1—C11—C6	117.67 (18)
N1—C8—H8A	109.7	C14^ii^—C12—C6	120.88 (17)
C10—C8—H8A	109.7	C14^ii^—C12—H12A	119.6
N1—C8—H8B	109.7	C6—C12—H12A	119.6
C10—C8—H8B	109.7	C12^ii^—C14—C6	121.00 (17)
H8A—C8—H8B	108.2	C12^ii^—C14—H14A	119.5
N1—C9—C13	110.28 (14)	C6—C14—H14A	119.5
N1—C9—H9A	109.6		

Symmetry codes: (i) −*x*−1, *y*, −*z*−1/2; (ii) −*x*, −*y*, −*z*−1.

### Hydrogen-bond geometry (Å, °)

**Table:**

*D*—H···*A*	*D*—H	H···*A*	*D*···*A*	*D*—H···*A*
N1—H1N1···O1^iii^	0.90	1.91	2.7958 (19)	169
N1—H2N1···O2	0.90	1.82	2.7213 (18)	174
N1—H2N1···O1	0.90	2.61	3.1528 (19)	120

Symmetry codes: (iii) −*x*−1/2, −*y*+1/2, −*z*−1.
